# Air Quality in a Dental Skills Lab during the SARS-CoV-2 Pandemic: Results of an Experimental Study

**DOI:** 10.1155/2022/9973623

**Published:** 2022-06-24

**Authors:** Christian Graetz, Naomi Sayk, Paulina Düffert, Ralf Heidenreich, Christof E. Dörfer, Miriam Cyris

**Affiliations:** ^1^Clinic for Conservative Dentistry and Periodontology, University Hospital Schleswig-Holstein, Campus Kiel, Arnold-Heller-Str. 3, Haus B, Kiel 24105, Germany; ^2^Institute of Air Handling and Refrigeration, Bertolt-Brecht-Allee 20, Dresden 01309, Germany

## Abstract

**Objectives:**

The study aimed to analyze different ways to control air quality during/after aerosol-generating procedures (AGPs) in a small skills lab with restricted natural air ventilation in preclinical dental training (worst-case scenario for aerogen infection control). Different phases were investigated (AGP1: intraoral high-volume evacuation (HVE); AGP2: HVE plus an extraoral mobile scavenger (EOS)) and afterward (non-AGP1: air conditioning system (AC), non-AGP2: AC plus opened door).

**Methods:**

Continuous data collection was performed for PM1, PM2.5, and PM10 (µg/m^3^), CO2 concentration (ppm), temperature (K), and humidity (h^−1^) during two summer days (AGP: *n* = 30; non-AGP: *n* = 30). While simulating our teaching routine, no base level for air parameters was defined. Therefore, the change in each parameter (Δ = [post]-[pre] per hour) was calculated.

**Results:**

We found significant differences in ΔPM2.5 and ΔPM1 values (median (25/75th percentiles)) comparing AGP2 versus AGP1 (ΔPM2.5: 1.6(0/4.9)/−3.5(−10.0/−1.1), *p*=0.003; ΔPM1: 1.6(0.6/2.2)/−2.2(−9.3/−0.5), *p*=0.001). Between both non-AGPs, there were no significant differences in all the parameters that were measured. ΔCO_2_ increased in all AGP phases (AGP1/AGP2: 979.0(625.7/1126.9)/549.9(4.0/788.8)), while during non-AGP phases, values decreased (non-AGP1/non-AGP2: −447.3(−1122.3/641.2)/−896.6(−1307.3/−510.8)). ∆Temperature findings were similar (AGP1/AGP2: 12.5(7.8/17.0)/9.3(1.8/15.3) versus non-AGP1/non-AGP2: −13.1(−18.7/0)/−14.7(−16.8/−6.8); *p* ≤ 0.003)), while for ∆humidity, no significant difference (*p* > 0.05) was found.

**Conclusions:**

Within the limitations of the study, the combination of HVE and EOS was similarly effective in controlling aerosol emissions of particles between one and ten micrometers in skill labs during AGPs versus that during non-AGPs. After AGPs, air exchange with the AC should be complemented by open doors for better air quality if natural ventilation through open windows is restricted.

## 1. Introduction

It is indisputable that dentistry involves many noninvasive or invasive activities, often associated with droplets and aerosols. These conditions are commonly regarded as a potential infection risk for the whole dental team [1]. Therefore, adequate protective measures against pathogens transmitted via droplets, splatters, or aerosols from patients' oral cavities are of high importance during all aerosol-generating procedures (AGPs) in dentistry [2, 3]. Particularly, limited data are available for the treatments themselves; realistic simulations of mechanical treatment situations have been conducted, but the interaction with breathing patients has not been investigated yet. Furthermore, the special situation of dental teaching (e.g., additional supervisors and longer treatment time) has rarely been investigated and is still affected by many unknown factors regarding the ongoing SARS-CoV-2 pandemic [4]. Careful consideration is required as education needs to be continued, but simultaneously, students, teachers, and supervisors need to be protected against infections. As a result of sending home students and pausing patient care during the pandemic, concerns about the safety, patient care, and clinical competence of students have arisen [4, 5].

Therefore, in the previous study [6], we investigated a situation in preclinical dental teaching courses and found that a routinely utilized high-volume evacuation (HVE) system for intraoral suction plus an additionally used mobile extraoral scavenging device (EOS) effectively reduced the emitted smaller particle matter (PN: 0.1–0.3 *μ*m) during AGP. No benefit was found for particles >0.3 *µ*m up to 5 *µ*m. This is in line with other studies [7, 8]. However, we must stress that this situation simulated by manikin heads differs from that used to represent the oral cavity. The findings cannot provide sufficient insight to understand bioaerosol infection risk [9]. Furthermore, there is still a controversy about the proportion of different routes of droplets or airborne transmission [10, 11] that are responsible for virus spread indoors [12].

Various measures are recommended to prevent the transmission of germ and viruses in dentistry [13]. These measures can be implemented (1) naturally (e.g., ventilation with fresh air through open windows/doors) or (2) supported by technical systems (e.g., air conditioning system (AC), fixed room ventilation system, or EOS). Principally, cross-ventilation with an air change rate up to 40 (ACH: amount of air supplied in one hour per room) is considered to be the most effective method [14]. However, due to unfavorable room architecture and low temperature differences, the effectiveness of natural ventilation can also decrease significantly (ACH: 0.3–1.5) [14]. Thus, there are various variables that influence the ACH (e.g., the number of windows and doors, seasons, and specific treatment/exposure situations). Since the data from our skill lab on this were limited, we aimed to measure the ΔPM for air exchange with AC only (non-AGP1) versus air exchange through AC plus an open door (non-AGP2) in a worst-case scenario (a high number of persons inside a small room, hot summer, and restricted natural air ventilation) for SARS-CoV-2 infection after simulating different AGPs (tooth preparation, air polishing, and ultrasonic scaling) during preclinical student training settings. The hypothesis to be tested was that non-AGP2 will be more effective in reducing particle matter and improving air quality after AGPs than non-AGP1.

## 2. Materials and Methods

This article does not contain any studies with human participants or animals performed by any of the authors. For this type of study, formal consent was not needed.

### 2.1. Experimental Setup: Manikin Head and Test Dental Procedure

The study set simulated student treatment of a manikin head (Kavo, Biberach, Germany) as part of the education in a preclinical study section to avoid unnecessary risks to operators due to the SARS-CoV-2 pandemic situation. The aerosol particles were measured in vitro in the categories of PM1 *µ*m, PM2.5 *µ*m, and PM10 *µ*m generated by different AGPs in a small skills lab (room volume 47.23 m^3^; room length/width/height: 4.81 m/3.34 m/2.94 m; and windows cannot be opened). During the AGP phase, neither natural nor technical air ventilation was performed for reproducible conditions. Four investigators were inside the room; two served as students (treating), one served as the supervisor, and one served as the person responsible for the measurement technique. All procedures were performed according to internal guidelines for preclinical training (date: summer 2021), including that all investigators wore a surgical mask at all times (3M Deutschland GmbH, Neuss, Germany).

### 2.2. Aerosol-Generating Procedures (AGP) and High-Volume Evacuation (HVE)

Phases of AGP (*n* = 30) were simulated either high-speed tooth preparation with a contra-angle handpiece at 200000 rpm (Kavo, Biberach, Germany) or two different procedures of professional tooth cleaning with an air polishing device (LM-Instruments Oy, Pargas, Finland) and scaling with an ultrasonic scaler (Kavo, Biberach, Germany) for supragingival biofilm and calculus removal over eleven minutes. Prior to each test, the flow rate was calibrated in line with the manufacturer's specifications (high-speed tooth preparation: 50 ml/min; ultrasonic scaler: 30 ml/min; and air-polishing device: 20–40 ml/min).

During all AGPs, a high-volume suction cannula with a diameter of 16 mm and a saliva ejector were used in combination with a clinical internal HVE for reproducible conditions at a flow rate of approximately 300 l/min (Dürr, Bietigheim-Bissingen, Germany).

In addition, in half of the tests (AGP2), an HVE system was combined with a mobile extraoral scavenging device (EOS) (JakAir Mobile System, ULT, Löbau, Germany). The EOS was equipped with a ULPA-U15 filter (ULT, Löbau, Germany). The intake of the particle counter (Aerosol spektrometer Mini WRAS, GRIMM Aerosol Technik Ainring GmbH & Co. KG, Ainring, Germany) and the EOS were both placed at the same level and a distance of 0.35 m on the left front side of the manikin head.

Randomized follow-up of the treatment procedures in AGP (high-speed tooth preparation or an ultrasonic scaler or an air-polishing device with HVE versus high-speed tooth preparation or an ultrasonic scaler or an air-polishing device with HVE + EOS) and afterward non-AGP phases (AC alone versus AC + open door) was performed using computer-generated random numbers (Microsoft Excel 16, Microsoft Corporation, One Microsoft Way Redmond, WA, USA).

### 2.3. Nonaerosol-Generating Procedures (Non-AGP) and the Air Conditioning System (AC)

Conversely, according to the AGPs, during non-AGPs (*n* = 30), the air conditioning system (AC) was always switched on. In non-AGP2, the AC was added by the opened door. For all non-AGPs, no restriction of time duration was made. Each non-AGP was individually stopped, and the time was measured when all necessary pre/postactivities for AGPs (e.g., organization of new instruments, refilling of material, surface disinfection, transport of used instruments outside the room, etc.) ended.

### 2.4. Particle Measurements

Measurement of the particle number concentration (counts/m^3^) was continuously performed by a particle counter (Aerosol spektrometer Mini WRAS, GRIMM Aerosol Technik Airing GmbH & Co. KG, Ainring, Germany). The particle counter measured the concentration of particles in three categories of PM1 (1 *µ*m), PM2.5 (2.5 *µ*m), and PM10 (10 *µ*m). The logging interval was 1 minute. To keep track of the air conditions in the room, a single-beam and dual-wavelength nondispersive infrared device (CARBOCAP, Vaisala Oy, Helsinki, Finland) was used to measure CO_2_ concentrations, room temperature, and relative air humidity.

### 2.5. Outcomes

Due to the aim of investigating the concentration of particles in routine preclinical training situations in our dental school, we performed all tests in a skills lab to simulate a worst-case scenario for SARS-CoV-2 infection (a high number of persons inside a small room, a higher number of AGPs, summer, windows cannot be opened) and for the high reproducibility of our in vitro setting. Moreover, we defined no normal air level for particle evaluation. Therefore, ΔPM (ΔPM = [post-PM]-[pre-PM]*µ*g/m3 per hour) and ΔCO_2_ (ΔCO_2_ = [post-CO_2_]-[pre-CO2]ppm per hour) were calculated for different observation phases. Moreover, we calculated the change in room temperature (∆temp = [post-temp]-[pre-temp]K per hour) and the change in the relative air humidity (∆hum = [post-hum]-[pre-hum]% per hour). The time duration of all AGPs was similar (11 min), and the time duration of non-AGPs was not consistent as mentioned before. Therefore, a calculation of all parameters per hour was performed for better comparability.

### 2.6. Statistical Analysis

The determined data of the particle counter and the single-beam and dual-wavelength nondispersive infrared device could be extracted in such a way that a direct entry in Microsoft Excel (Microsoft Excel 16, Microsoft Corporation, One Microsoft Way Redmond, WA, USA) was possible. No sample calculation was performed before investigation. Subsequently, the data sheets were integrated into SPSS Statistics (SPSS Statistics 27, IBM, Chicago, IL, USA) for statistical analysis. A test for normal distribution was performed using Kolmogorov–Smirnov and Shapiro–Wilk tests; no normal distribution was observed. Therefore, a comparison of means was performed using the Kruskal–Wallis test with Bonferroni correction for multiple tests to detect significant differences according to ΔPM and ΔCO_2_ values among the different observation phases (AGP1/non-AGP1 and AGP2/non-AGP2, respectively). All tests were two-sided; statistical significance was assumed if *p* ≤ 0.05.

## 3. Results

**Figure 1:**
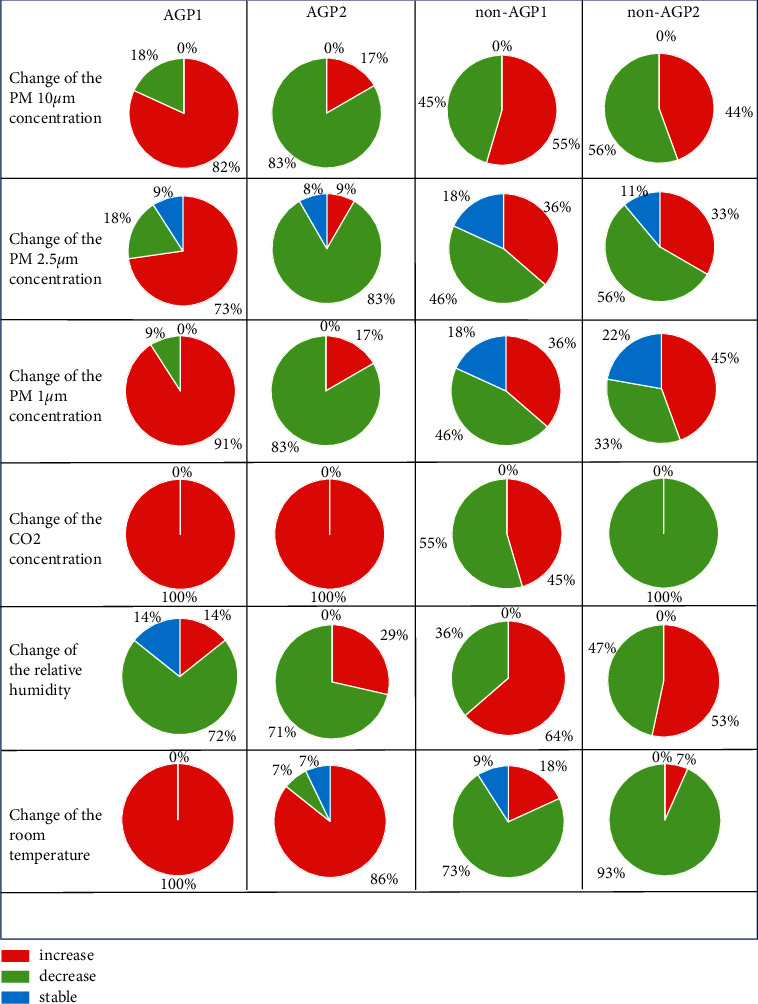
Illustration of the distribution of trends (increasing, stable, and decreasing) per test of AGP versus non-AGP for ΔPM10, ΔPM2.5, ΔPM1, ΔCO_2_, ∆humidity, and ∆temperature per hour. CO_2_: carbon dioxide concentration; AGP1: aerosol generated procedure with intraoral high-volume evacuation (HVE); AGP2: aerosol-generated procedure with HVE and extraoral mobile extraoral scavenger; non-AGP1: no aerosol-generated procedure under conditions of technical ventilation; non-AGP2: no aerosol-generated procedure under conditions of open windows; hum: relative humidity; temp: room temperature.

Contrary to AGP2, the majority of all AGP1 trials (73%–91%) resulted in an increasing trend of particle matter in the room (Figure 1). According to the change in PM2.5 and PM1 values (ΔPM: median (25/75th percentiles)), for AGP2 versus AGP1, negative results were measured (ΔPM2.5 : 1.6 (0/4.9) *µ*g/m^3^ per hour/−3.5 (−10.0/−1.1 *µ*g/m3 per hour), *p*=0.003; ΔPM1: 1.6 (0.6/2.2) *µ*g/m^3^ per hour/−2.2 (−9.3/−0.5) *µ*g/m3 per hour, *p*=0.001). For smaller particles of 1 *µ*m in 83% of all AGP2, a decrease in ΔPM1 was measurable (Figure 1). In the category of larger particle sizes of 10 *µ*m, no significant differences in ΔPM among all observation phases were detectable (all AGPs vs. non-AGPs, AGP1 vs. AGP2, non-AGP1 vs. non-AGP2: *p* > 0.05; Table 1).

Moreover, between both non-AGPs (time duration: minimum 4 min/maximum 19 min), no significant difference for all ΔPM categories was detected (*p* > 0.05, Table 1). The particle emission decreased by air ventilation in nearly 33%–56% of all non-AGPs or was stable in 11%–22% at the same PM level in all three categories of the particle size (Figure 1). Therefore, we found an increasing trend for particle matter in 36%–55% of all non-AGPs in the room.

We measured the CO_2_ concentration for all AGP phases always with an increasing trend (Figure 1) but found no significant difference between AGP1 and AGP2 for the values of ΔCO_2_ (AGP1/AGP2: 979.0(625.7/1126.9) ppm per hour/549.9(4.0/788.8) ppm per hour; *p* > 0.05; Table 1). Interestingly, for all non-AGP2 trials, we found a decreasing trend for CO_2_ room concentration and a significant difference in the values of ΔCO_2_ compared to both AGP phases (−896.6(−1307.3/−510.8) ppm per hour, *p* ≤ 0.011; Table 1). The ΔCO_2_ values in non-AGP1 also decreased in 55% of all trials, however, with a smaller ΔCO_2_ value of −447.3(−1122.3/641.2) ppm per hour (non-AGP1 versus non-AGP2: *p* > 0.05).

We found no significant difference in ∆hum for AGPs versus non-AGPs or intragroup AGP and non-AGP (Table 1). For AGP1, the room temperature increased by 12.5 (7.8/17.0) K per hour (AGP2: 9.3 (1.8/15.3 K per hour); *p* > 0.05) and decreased by −13.1 (−18.7/0) K per hour in non-AGP1 (non-AGP2: −14.7 (−16.8/−6.8) K per hour; *p* > 0.05). However, between all different observation phases, significant differences could be measured (Table 1).

## 4. Discussion

It is indisputable that we must protect students and dental staff against airborne infection during all teaching courses. Since the advent of the SARS-CoV-2 pandemic, the topic has gained worldwide interest, and a variety of methods have been discussed to minimize pseudogene disease transmission [15]. In a systematic review of current data from Fiorillo et al. [16], it was found that COVID-19 can remain infectious for several hours in aerosols and even for several days on surfaces. Therefore, it is very important to minimize the infectivity and potential deposition of COVID-19 from aerosols onto surfaces by various measures, such as air exchange and disinfection [16]. During preclinical dental training with manikin heads, current results indicate that the addition of an EOS leads to significantly lower particle concentrations. This effect could be analyzed for particles with a diameter up to 2.5 *µ*m (Table 1, *p* < 0.001), which is in line with former investigations [6–8]. However, conditions in skills labs differ from treatment situations in dental practice, e.g., social distancing is limited, and a high number of humans have to be managed routinely during education classes. For instance, the student's stress level, especially in examiners, will significantly influence the room temperature, humidity, and CO_2_ level. Air conditions should be held within the recommended guidelines to provide a good learning environment, which is crucial for positive learning outcomes and additionally consider the hygiene standards in teaching during the pandemic. In addition, protective procedures could be limited, as, e.g., natural ventilation with open windows of the room is restricted (our scenario). In particular, hands-on instruction in the skills lab can only be done in person and is absolutely necessary for good dental education. One way to increase student and staff safety would be to have preventive triage. This could be accomplished by asking for symptoms of COVID-19 before entering the skills lab or by establishing a well-communicated policy so that all persons with typical symptoms stay home, similar to the procedure for patients [17].

### 4.1. Non-AGP: How can Air Ventilation be Organized in the Skills Lab?

We found comparable decreasing particles per hour during technical air ventilation in both non-AGPs. However, additional open doors during non-AGP phases showed trends for better air exchange as measured by the CO_2_ and temperature levels (Table 1; *p* > 0.05). When air conditioning was added by the open door during the breaks, the CO_2_ concentration decreased during all times, and the temperature decreased in 93% of all non-AGP2 phases. This helps to improve the air quality after AGPs with increasing temperature caused by the human and technical equipment associated with decreasing humidity. Physically, if the temperature increases, the relative humidity consequently decreases and vice versa. The change in humidity was without significant differences between all observation phases; however, we identified significant temperature changes between AGPs versus non-AGP (Table 1). With the help of air ventilation in non-AGP phases, the temperature decreased significantly more than the increasing temperature during both AGPs (*p* < 0.001). This showed the high efficiency of air ventilation, independent of the type of technical or natural, and will significantly impact the infection processes; for example, viruses are more likely to stay infectious when the air humidity is high at lower room temperatures, and therefore, the liquid phase does not vaporize [18].

Unfortunately, a limitation of our experimental setup is that for practical reasons, no baseline value before each AGP was calibrated. In compensation, the differences in all parameters (at per hour) instead of absolute values were analyzed. Nevertheless, the relatively high ∆CO_2_ and ∆temp in our investigation are worth mentioning, as they may indicate a high amount of exhaled air in the room, as discussed in our previous study [6]. The decisive factor here is that every person in the skills lab wore a certified surgical mask according to the EN 14683 standard at all times, providing a barrier to particles but not CO_2_ gas. According to the results by Kun-Szabo et al. [19], we could assume that a non-AGP time span of approximately nine minutes with technical air conditioning plus conventional airing through doors (non-AGP2) leads to a sufficient amount of fresh air and reduces the particle concentration in the room. In contrast, through open doors and windows, e.g., dust could be carried inside and lead to an increased concentration of larger particle sizes [2]. According to the negative pressure inside our skills lab (dust from the corridor was sucked inside), we measured such a phenomenon in the current study for PM10 caused by a construction site on another floor. It can be hypothesized that these larger particles play only a minor role in disease transmission.

### 4.2. AGP-HVE and EOS: How to Control Aerosol Emission in Skills Lab?

During AGP, the use of an HVE suction system with high-flow cannulas (boring diameter >10 mm) eliminates particles with a diameter >5 *µ*m [20,21]. Furthermore, due to their higher weight, larger particles fall rapidly, and potential infection risks can easily be managed with adequate surface disinfection [13]. Smaller particles (<5 *µ*m) were less affected by dental suction due to their smaller surface areas and/or higher speed and therefore remained in the air for a longer time. The problem is that these smaller particles are likely to be highly infectious, as the infectivity of virus-laden particles correlates with their size. The smaller the particles are, the more likely they are to transmit disease. For instance, the coronavirus RNA genome was detected in 40% of aerosol particles smaller than 5 *µ*m but only in 30% of larger particles (>5 *µ*m) [22]. In clinical cases, many of these particles are aerosolized and contain a large amount of kinetic energy (e.g., high-speed tooth preparation or air polishing) [23].

It must be emphasized that infection control recommendations for dental practices [24] cannot be transferred 1:1 in dental teaching programs. Nevertheless, combining strategies of protective procedures, including wearing masks, the use of HVE, or airing rooms, seem mandatory during dental treatments in student courses [25]. For classes teaching dental procedures in skills labs, general infection control methods, such as personal protective equipment (e.g., face masks) and physical distancing, are crucial elements to protect students and teachers [5]. Some might argue that dental teaching classes could be held online. Partially, this might be an alternative, but clearly, learning practical dental skills online is facing challenges [26]. Therefore, whenever protective procedures are limited, especially natural ventilation with open windows of the room as in our skills lab [27], a mobile EOS with an HEPA filter (high efficiency particulate air) could help to reduce the particle concentration further and faster, as indicated by our current data. We calculated an ACH of approximately 3 h^−1^ for the tested mobile EOS device (all particle sizes), which is comparable with other study results [8,28]. However, it is nearly doubled compared with the calculated ACH of approximately 1.8 h^−1^ in our skills lab. This is acceptable according to the recommendation of at least 2 h^−1^ by a statement of the German Society of Hospital Hygiene (DGKH). We could assume that if both technical systems (AC + EOS) are utilized in addition to the mandatory HVE, a theoretical ACH up to 5 h^−1^ is possible, which is described as necessary to provide a safer working environment [29]. Hence, our tested mobile EOS device works according to the recirculation principle, which means that even in continuous operation, only a fraction of the room air is cleaned. Consequently, in a larger skills lab with a high turnover rate, generously dimensioned units are necessary. However, a higher turnover rate might lead to higher noise pollution during AGP trainings [7,30] and cost-intensive operations [31]. In total, the acquisition and maintenance costs of an EOS device should not be underestimated, as they require professional installation and regular changes of the potentially contaminated filters [27,30]. On the other hand, opening a window is an easy, cheap, and yet effective way to reduce floating airborne particles with air ventilation [32]. The effectiveness of ventilation through open windows varies widely depending on the weather and other factors. Therefore, before acquisition of an EOS device for routine use in dental schools, all of the above advantages and disadvantages should be considered. In our estimation, EOS devices can represent a complementary measure for aerogene infection control in small- to medium-sized skills labs, which are always associated with inconsiderable acquisition and operating costs, noise, or limitation of space.

### 4.3. Advantages and Disadvantages of the Experimental Setup

First, the experimental setting allowed a high standardization and reproducibility of all performed procedures in AGP and non-AGP phases. Second, the simulation of the preclinical treatments was nearly without any restriction due to the method. Third, as many room variables were measured, the results could be transferred into other settings, e.g., to develop an air ventilation plan for the larger skills lab or restriction of the number of persons per room. As a weakness, the outlined results using particle sampling measurement could not be compared with other methodologies for measuring aerosolization in dentistry directly, e.g., biological air sampling [33], the culture of settle plates [34], and the detection of fluorescent markers via indirect techniques with coloring the fluid [20,35]. The used methodology of particle sampling was established [2,35]. However, for our setting, e.g., we could not measure the possible superimposed background particle movements, we did not set PM levels at the baseline [3]. To overcome this, we analyzed only the change in particles per hour to reduce the possible impact of people moving inside the room as one factor that could result in higher air turbulence with higher resuspension of particle deposition on surfaces and the ground inside the room [36]. Furthermore, we monitored the air velocity near the point of air sampling (air samplers using a small air vent for particle sampling) and found an average air velocity comparable to natural thermal effects in all tests [6]. It is also an in vitro investigation in which no infectious aerosol was generated. Therefore, the risk of infection in this regard was not examined.

However, all previously mentioned limitations and restrictions of any experimental study should be taken into account when the current findings are used for internal guideline planning according to dental teaching in the skills labs (including acquisition of equipment or developing a room air ventilation plan, limitation of the number of persons per room, or duration of the course).

## 5. Conclusions

Within the limitations of the present experimental in vitro study (without infectious aerosols), to simulate dental students' training set up in a small skills lab with limited natural air ventilation through open windows, the technical method of air ventilation supplemented by an open door leads to better air quality after aerosol-generating procedures. Moreover, we found that for aerosol-generating procedures in our training and room setting, intraoral high-volume evacuation (HVE) and an extraoral mobile scavenger (EOS) should be mandatory.

## Figures and Tables

**Table 1:** . Results of ΔPM (ug/m3), ΔCO_2_ concentration (ppm), time duration (in minutes), Δtemperature (K), and ∆humidity (%) during different observation phases per hour.

N of valid test (invalid)	AGP1	AGP2	Non-AGP1	Non-AGP2	APG2 vs. non-APG1	APG2 vs. non-APG2	APG2 vs. APG1	Non-APG1 vs. non-APG2	Non-APG1 vs. APG1	non-APG2 vs. APG1
	11 (4)	12 (2)	11 (4)	9 (6)						
ΔPM10	6.0 (1.1/10.9)	−2.2 (−12.0/4.2)	0.3 (−12.7/14.6)	−1.6 (−6.9/4.0)	*p* > 0.05	*p* > 0.05	*p* > 0.05	*p* > 0.05	*p* > 0.05	*p* > 0.05
ΔPM2.5	1.6 (0/4.9)	−3.5 (−10.0/−1.1)	0 (−10.0/1.7)	−1.5 (−3.3/1.3)	*p* > 0.05	*p* > 0.05	*p*=0.003	*p* > 0.05	*p*=0.037	*p* > 0.05
ΔPM1	1.6 (0.6/2.2)	−2.2 (−9.3/−0.5)	0 (−7.3/2.2)	0 (−2.0/0.7)	*p* > 0.05	*p* > 0.05	*p*=0.001	*p* > 0.05	*p* > 0.05	*p* > 0.05
ΔCO_2_	979.0 (625.7/1126.9)	549.9 (4.0/788.8)	−447.3 (−1122.3/641.2)	−896.6 (−1307.3/−510.8)	*p* > 0.05	*p*=0.011	*p* > 0.05	*p* > 0.05	*p* < 0.025	*p* < 0.001
∆temp	12.5(7.8/17.0)	9.3(1.8/15.3)	−13.1(18.7/0)	−14.7(−16.8/−6.8)	*p*=0.003	*p* < 0.001	*p* > 0.05	*p* > 0.05	*p* < 0.001	*p* < 0.001
∆hum	−9.0(−14.0/0)	−4.1(−11.9/1.6)	3.4(−12.7/26.4)	−2.2(−8.0/9.1)	*p* > 0.05	*p* > 0.05	*p* > 0.05	*p* > 0.05	*p* > 0.05	*p* > 0.05
Time duration	11 (11/11)	11 (11/11)	8.0 (5.0/16.0)	9.0 (5.0/10.0)	*p* > 0.05	*p*=0.029	—	*p* > 0.05	*p* > 0.05	*p*=0.037

Kruskal–Wallis test with Bonferroni correction for multiple tests; significant *p* ≤ 0.05 CO_2_: carbon dioxide concentration; AGP1: aerosol generated procedure with intraoral high-volume evacuation (HVE); AGP2: aerosol-generated procedure with HVE and an extraoral mobile extraoral scavenger; non-AGP1: no aerosol-generated procedure under conditions of technical ventilation; non-AGP2: no aerosol-generated procedure under conditions of open windows; hum: relative humidity; temp: room temperature.

## Data Availability

Data are available from the corresponding author for researchers who meet the criteria for access to confidential data.
